# MiR-217 Regulates SIRT1 Expression and Promotes Inflammatory and Apoptotic Responses in Osteoarthritis

**DOI:** 10.3390/genes14122155

**Published:** 2023-11-29

**Authors:** Aliki-Alexandra Papageorgiou, Athanasios Roussos, Ioanna Papathanasiou, Charalampos Balis, Theophilos Karachalios, Sokratis E. Varitimidis, Konstantinos N. Malizos, Aspasia Tsezou

**Affiliations:** 1 Laboratory of Cytogenetics and Molecular Genetics, Faculty of Medicine, University of Thessaly, Biopolis, 41500 Larissa, Greece; alipapageorgiou@med.uth.gr (A.-A.P.); thanosrouss@gmail.com (A.R.); iopapat@uth.gr (I.P.); chbalis@gmail.com (C.B.); 2 Department of Biology, Faculty of Medicine, University of Thessaly, Biopolis, 41500 Larissa, Greece; 3 Department of Orthopedic Surgery, Faculty of Medicine, School of Health Sciences, University of Thessaly, Biopolis, 41500 Larissa, Greece; kar@med.uth.gr (T.K.); svaritimidis@ortho-uth.org (S.E.V.); malizos@med.uth.gr (K.N.M.)

**Keywords:** miR-217, SIRT1, osteoarthritis, inflammation, apoptosis

## Abstract

Previous studies have reported miR-217 uregulation in age-related pathologies. We investigated the impact of miR-217-5p on sirtuin 1 (SIRT1) regulation in human osteoarthritic (OA) chondrocytes. MiR-217 target enrichment analyses were performed using three public databases, Gene Ontology and Kyoto Encyclopedia of Genes and Genomes. MiR-217-5p expression levels were quantified in normal and OA chondrocytes. SIRT1 expression levels, nuclear factor kappa-B p65 subunit (NF-κBp65) and p53 acetylation levels, and expression levels of OA-related pro-inflammatory markers [tumor necrosis factor α (TNFα), interleukin 1β (IL-1β), IL-6], pro-apoptotic markers [Bax, pro-caspase 3, cleaved caspase 3] and matrix regulators [matrix metalloproteinase (MMP)-1, MMP-13, MMP-9, Collagen 2 (COL2A1), Aggrecan (ACAN)] were evaluated in miR-217 mimic-treated and/or miR-217 inhibitor-treated OA chondrocytes, with/without subsequent treatment with siRNA against SIRT1 (siSIRT1). MiR-217-5p was upregulated in OA chondrocytes, while target prediction/enrichment analyses revealed *SIRT1* as miR-217 target-gene. Deacetylation of NF-κBp65 and p53 in miR-217 inhibitor-treated OA chondrocytes was reversed by siSIRT1 treatment. MiR-217 inhibitor-treated OA chondrocytes showed increased COL2A1, ACAN and decreased IL-1β, IL-6, TNFα, Bax, cleaved caspase 3 and MMPs expression levels, which were reversed following miR-217 inhibitor/siSIRT1 treatment. Our findings highlight the impact of miR-217-5p on SIRT1 downregulation contributing to OA pathogenesis.

## 1. Introduction

Osteoarthritis (OA), the most prevalent type of arthritis, constitutes a considerable health burden in high income countries [1], with aging being among the most important OA risk factors [2]. The prevalence of radiographic OA shows an increasing trend with each decade of life, fluctuating from 33% among adults over 60 years to almost 45% over 80 years [3]. Although OA molecular mechanisms are not fully elucidated, cell senescence, gradual degeneration of cartilage extracellular matrix (ECM), inflammation of the synovium and aberrant apoptotic/survival patterns in OA chondrocytes have been reported as key players in OA progression [4,5,6,7].

MicroRNAs (miRNAs) orchestrate cellular homeostasis in multiple levels, mainly via the posttranscriptional control of gene networks [8]. They play crucial roles in age-related pathologies, such as cardiovascular disease (CVD) and hypertension [9], atherosclerosis [10], type 2 diabetes [11], neurodegenerative diseases such as Parkinson’s [12], and various types of cancer [13]. In OA, numerous studies have demonstrated miRNAs’ role in inflammation, apoptosis, ECM degeneration and chondrogenesis [14,15].

MiR-217-5p has been strongly correlated with aging and is among the highest induced miRNAs during endothelial cell senescence and dysfunction in cardiovascular disease (CVD) [16,17]. It has also been associated with macrophage apoptosis in atherosclerosis [18], and podocyte apoptosis in diabetic nephropathy [19] and has been investigated in various malignancies, such as liver cancer [20] and acute myeloid leukemia [21]. In addition, miR-217 inhibition has been associated with alleviation of Parkinson’s disease manifestations, such as inflammatory pathway signaling, excessive oxidative stress, as well as an increased rate of apoptosis in human neuroblastoma SH-SY5Y cells [22]. Nevertheless, the impact of miR-217 in OA pathology has not been elucidated yet.

Sirtuin 1 (SIRT1) is a member of the NAD+-dependent protein deacetylases family that has been previously described as a central regulator of aging, cellular metabolic functions, senescence, inflammation and apoptosis [23]. SIRT1 exhibits decreased expression in age-related chronic inflammatory pathologies, including OA [24,25,26]. MiR-217/SIRT1 interaction has been revealed in a number of studies to contribute to the progression of age-related pathological/morbid conditions involving oxidative stress such as CVD, atherosclerosis, diabetes, neurodegenerative diseases and cancer [27]. Indicatively, miR-217 has been correlated with deregulated macrophage apoptosis in an endothelium model for atherosclerosis in mice, where it was found to downregulate SIRT1 in vivo [17]. In Tohoku Hospital Pediatrics-1 (THP-1) acute monocytic leukemia after treatment of macrophages with oxidized low-density lipoprotein (ox-LDL) as an atherosclerotic cell model, miR-217 was found to target *SIRT1* affecting downstream inflammatory responses, contributing, thus, to atherosclerosis progression [18]. MiR-217/SIRT1 interaction has been studied in various types of cancer including hepatocellular carcinoma, osteosarcoma and colorectal cancer [28]. Nevertheless, the effect of miR-217/SIRT1 interaction on OA progression remains unaddressed.

In the context of this study, we aimed to address the impact of miR-217 on deacetylase’s SIRT1 expression and activity levels in OA human chondrocytes and consequently in OA incidence.

## 2. Materials and Methods

### 2.1. Patients and Sample Collection

Cartilaginous specimens were collected from 20 osteoarthritic patients (12F/8M; mean age 67.3 ± 8.2 years) who underwent total or partial knee replacement surgery. Ten (10) healthy participants (6F/4M; mean age 52.4 ± 6.9 years) without clinical history of arthropathy, or any clinical symptoms of OA, comprised the control group. All specimens were collected from the Department of Orthopedic Surgery of the University Hospital of Larissa, Greece. All OA cartilage tissue fragments were obtained from tibial plateaus and/or femoral condyles that exhibited severely degenerated cartilage lesions, with major or minor exposure of subchondral bone. Cartilaginous specimens from healthy donors were collected during amputation or fracture repair surgery. Radiographs of all patients were taken prior to surgery and were assessed regarding OA severity according to the Kellgren-Lawrence (K/L) grading scale. All OA patients were graded with a K/L score > 2. A blinded clinical evaluation of radiographs was conducted by two independent expert observers. Cases of autoimmune diseases, such as rheumatoid arthritis, as well as chondrodysplasias, and post-traumatic or septic OA were not included in the experimental group of our study.

### 2.2. Articular Chondrocytes’ Cultures

Cartilaginous specimens were dissected and digested with 1 mg/mL pronase plus 1 mg/mL collagenase P (Roche Applied Science, Mannheim, Germany). Next, chondrocytes isolated from cartilage tissue fractions of healthy individuals (normal) and OA patients (OA) were cultured in flasks, using Dulbecco’s Modified Eagle’s Medium/Ham’s F-12 (Thermo Fisher Scientific, Waltham, MA, USA). The culture medium was further supplemented with FBS (10%) and penicillin–streptomycin antibiotics (1%) (both by GIBCO, Thermo Fisher Scientific, Waltham, MA, USA) at 37 °C in a 5% CO_2_ humidified incubator, until reaching the desired confluence.

### 2.3. Prediction of miR-217-5p Target Genes and Functional Enrichment Analysis

The public databases TargetScan (https://www.targetscan.org/vert_80/ accessed on 7 September 2022), DIANA (microT) (https://dianalab.e-ce.uth.gr/microt_webserver/#/ accessed on 12 September 2022), miRDB (https://mirdb.org/ accessed on 11 September 2022), and miRTarBase (https://miRTarBase.cuhk.edu.cn/ accessed on 13 September 2022) were used for miR-217-5p target mRNAs prediction. Open Targets platform (https://www.opentargets.org/ accessed on 13 September 2022) was used to address miR-217-5p mRNA-targets that are involved in OA pathology. Next, ontology enrichment analysis for the predicted miR-217 targets was performed. The ontologies were enriched with biological processes and gene interaction regulatory pathways. Target enrichment analyses of Gene Ontology (GO) and Kyoto Encyclopedia of Genes and Genomes (KEGG) pathway enrichment analyses were carried out through the “enrichR” web-based tool (https://maayanlab.cloud/Enrichr/ accessed on 12 July 2023). Functions and/or pathways significantly enriched were identified based on the *p*-value < 0.05 criterion. The enrichment of top GO and KEGG pathways terms were visualized by dot plot analysis. Finally, the complementarity of miR-217-5p with its mRNA-target sequences was assessed via the TargetScan database.

### 2.4. Transfection of OA Chondrocytes with miR-217 Inhibitor and/or siRNA against SIRT1, or miR-217 Mimic

OA chondrocytes were seeded onto 6-well plates and counted at the desired density (3 × 10^5^ cells/well). One day after seeding, the culture medium was withdrawn and cells were treated with 50 pmol of Ambion™ mirVana™ miR-217-5p inhibitor and/or 100 pmol of Ambion™ siRNA against SIRT1, or 50 pmol of Ambion™ mirVana™ miR-217-5p mimic (all by Thermo Fisher Scientific, Waltham, MA, USA) for 48 h. Ambion™ mirVana™ miRNA inhibitor Negative Control #1 #4464076, Ambion™ mirVana™ miRNA mimic Negative Control #1 #4464058, and Ambion™ Silencer Negative Control #1 siRNA, #440402 (all by Thermo Fisher Scientific, Waltham, MA, USA) were added as negative controls. Lipofectamine™ RNAiMAX reagent (Thermo Fisher Scientific, Waltham, MA, USA) was used as a transfection medium, according to the protocol provided by the manufacturer. Following transfection experiments, chondrocytes, as well as cell culture medium were collected, while cell pellets were subjected to RNA and protein extraction in order to be used in further experimental procedures.

### 2.5. RNA Extraction, cDNA Synthesis and Quantitative Real-Time PCR (qRT-PCR)

First, total RNA was isolated from articular chondrocytes with TRIzol reagent (Thermo Fisher Scientific, Waltham, MA, USA). For complementary DNA (cDNA) synthesis, reverse transcriptase SuperScript III (Thermo Fisher Scientific, Waltham, MA, USA) was used. For quantitative amplification of miRNAs, the target cDNA was lengthened with a highly stable stem-loop primer designed for each miRNA specifically, during first-strand cDNA synthesis [29]. Quantification of miR-217-5p, SIRT1, tumor necrosis factor α (TNFα), interleukin-1β (IL-1β), IL-6, matrix metalloproteinase (MMP)-1, MMP-13, MMP-9, type II collagen (COL2A1) and aggrecan (ACAN) expression was performed on the ABI 7300 system (Applied Biosystems, Bedford, MA, USA), while 7300 system SDS software v.1.4.0 was used for results’ analysis. The 10 μL reaction mixture was prepared using 5 μL of Power SYBR Green PCR Master Mix (Thermo Fisher Scientific, Waltham, MA, USA), 500 nM of each primer, and 2 μL of cDNA. Amplification cycling included 45 cycles of; 95 °C (10 min), 95 °C (15 s) and 60 °C (1 min). The Integrated DNA Technologies (IDT) PrimerQuest™ Tool which incorporates Primer3 software v.2.2.3 (https://www.idtdna.com/pages/tools/primerquest, accessed on 12 July 2023) and OligoAnalyzer™ Tool v.3.1 (http://scitools.idtdna.com/analyzer/Applications/OligoAnalyzer/, accessed on 12 July 2023) were used to design the oligonucleotide and primers used in PCR amplification. All primer sequences are presented in Table 1. U6 small nuclear RNA (snRNA) and glyceraldehyde 3-phosphate dehydrogenase (GAPDH) internal control were used for normalization of miRNAs and mRNAs expression, respectively. Quantification of relative gene expression was conducted using the 2^−ΔΔCt^ method, according to Livak et al. 2001 [30].

### 2.6. Western Blot

Isolated chondrocytes’ pellets were diluted and subsequently lysed in RIPA buffer (150 mM NaCl, 10 mM Tris (pH 7.5), 1% Triton X-100, 1% Sodium Deoxycholated, 0.1% SDS and 1 mM EDTA) with the addition of protease inhibitors (Roche Applied Science, Mannheim, Germany). A Qubit^®^ fluorometer with the Qubit^®^ Protein Assay Kit (Thermo Fisher Scientific, Waltham, MA, USA) was used for protein concentration quantification. Protein samples of 20–50 μg were evaluated in 8–12% SDS–PAGE gel electrophoresis according to the standard protocol and subsequently blotted to a polyvinylidene difluoride (PVDF) membrane (Millipore, Billerica, MA, USA). For membrane blocking, 5% *w/v* non-fat dry milk diluted in PBS/0.1%Tween20 was used. Next, membranes were incubated overnight with an optimal dilution of the respective primary antibody, in blocking buffer at 4 °C. Anti-SIRT1 (1:1000, C14H4 Rabbit mAb #2496T; Cell Signaling Technology, Danvers, MA, USA), anti-Acetylated-p53 (anti-Acetyl-p53 (Lys382); 1:500, Rabbit, #2525; Cell Signaling Technology, Danvers, MA, USA), anti-Acetylated-Nuclear Factor kappa-B p65 subunit (anti-AcNF-κBp65 (Lys310); 1:500, Rabbit, #3045S; Cell Signaling Technology, Danvers, MA, USA), anti-BAX (1:200, (B-9): sc-7480 Mouse, Santa Cruz Biotechnology Inc., Dallas, TX, USA), anti-Caspase 3 (1:200, (E-8): sc-7272 Mouse, Santa Cruz Biotechnology Inc., Dallas, TX, USA) and anti-Cleaved Caspase 3 (anti- Cleaved Caspase 3 (Asp175), 1:250, Rabbit, #9661; Cell Signaling Technology, Danvers, MA, USA) were the antibodies used. Anti-β-actin (1:3000, (C4): sc-47778 Mouse, Santa Cruz Biotechnology Inc., Dallas, TX, USA) was used as the control for protein quantification. Membranes were, next, incubated with horseradish peroxidase (HRP)-conjugated secondary antibodies (anti-Rabbit; 1:10,000, SKU: BA1054-1 and anti-Mouse; 1:10,000, SKU: BA1050-1; Boster Biological Technology, Pleasanton, CA, USA), for 1 h at room temperature (RT). ECL substrates (Thermo Fisher Scientific, Waltham, MA, USA) were used in the protein band visualization step, while Uvitec Cambridge Chemiluminescence Imaging System was used for enhanced chemiluminescence detection. Representative immunoblots are presented in the respective figures, as blots were originally performed in triplicate. Protein expression was quantified via the ImageJ software v.1.53a (https://imagej.nih.gov/ij/, accessed on 12 July 2023), normalized relative to the housekeeping protein (β-actin).

### 2.7. Enzyme Linked Immunosorbent Assay (ELISA)

After treatments with miR-217 inhibitor and/or siRNA against SIRT1, negative inhibitor, miR-217-mimic or negative mimic, supernatants were collected via gentle centrifugation. Concentrations of TNFα, IL-1β and IL-6 were evaluated using Human TNFα (EΚ0525), Human IL-1β (EΚ0392) and Human IL-6 (EΚ0410) ELISA kits (Boster Biological Technology, Pleasanton, CA, USA) following the instructions provided by the manufacturer. The four-parameter logistic (4-PL) curve-fit was applied for the calculation of TNFα, IL-1β and IL-6 concentration in cell supernatants.

### 2.8. Statistical Analysis

Data analysis was operated via the SPSS 25.0 software. Statistical significance declared by the criterion of a *p*-value < 0.05 was determined using the appropriate tests; Mann–Whitney U-test and paired/independent samples *t*-test. Results are presented as mean ± standard error. Cell treatments were conducted in triplicate, with a minimum of three (3) individual OA samples, unless otherwise stated.

## 3. Results

### 3.1. Cellular miR-217-5p Target Genes Are Involved in Aging and OA-Related Pathways

**Figure 1:**
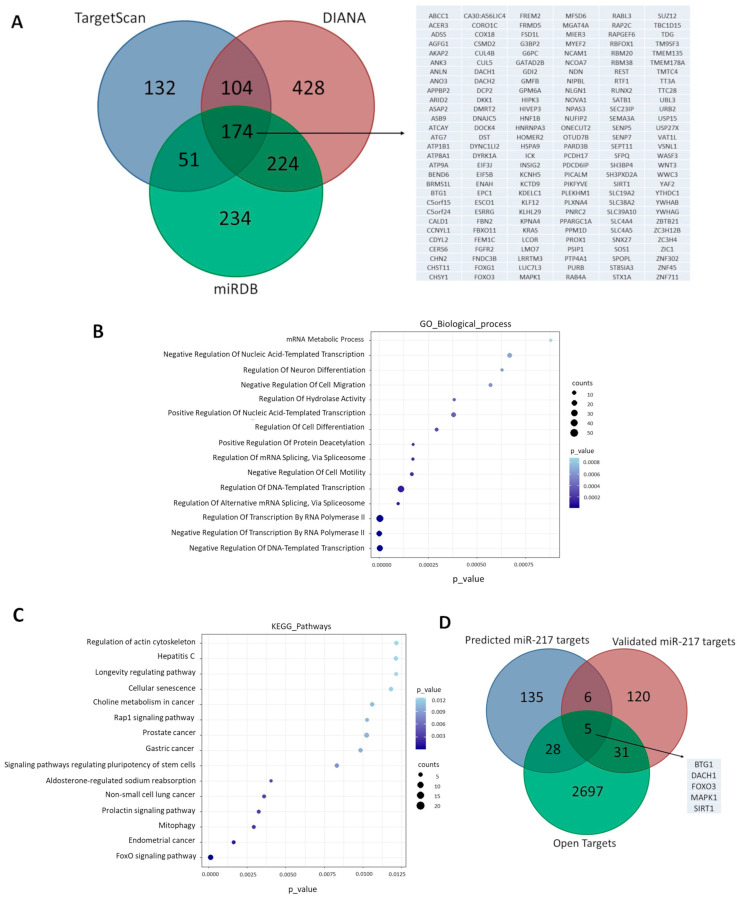
Cellular miR-217-5p target genes are involved in aging and OA-related pathways. (**A**) Venn diagram representing 174 common entries between TargetScan, DIANA (microT) and miRDB. Gene names are listed in alphabetical order. (**B**) GO biological process terms and (**C**) KEGG pathways of the 174 predicted miR-217 target genes visualized by dot plot. (**D**) Venn diagram representing five common entries between the 174 predicted miR-217 target genes, validated miR-217 targets acquired via miRTarBase, and OA-related miR-217 gene-targets acquired via Open Targets platform. Gene names are listed alphabetically.

To investigate possible pathways in which miR-217-5p is involved, we performed target prediction using the databases TargetScan, miRDB and DIANA (microT). Only the putative target mRNAs predicted in all three public databases were taken into consideration. We predicted 174 cellular genes to interact with miR-217-5p using the above criterion (Figure 1A). We next performed enrichment analysis with GO and KEGG to identify biological processes and cellular pathways affected by the identified targets. The analysis showed enrichment of specific cellular pathways related to gene expression regulation, protein/histone deacetylation, cell senescence and longevity, suggesting that miR-217-5p target genes are predominantly involved in molecular mechanisms associated with aging (Figure 1B,C, Supplementary Table S1). Next, we used the miRTarBase database to sort out the experimentally validated miR-217-5p mRNA targets. We found that 11 out of the 174 genes were validated miR-217-5p targets. Next, the Open Targets platform revealed that 5 out of the 11 miR-217-5p gene targets were involved in OA pathology (Figure 1D). Among them, SIRT1, a longevity factor with anti-aging and chondroprotective properties [31,32,33] was chosen for further experimentation. SIRT1 is one of the most robust miR-217 target genes in the literature, which we have previously found to be underexpressed in OA chondrocytes [34].

### 3.2. Differential Expression of miR-217 Affects SIRT1 Expression in OA Chondrocytes

**Figure 2:**
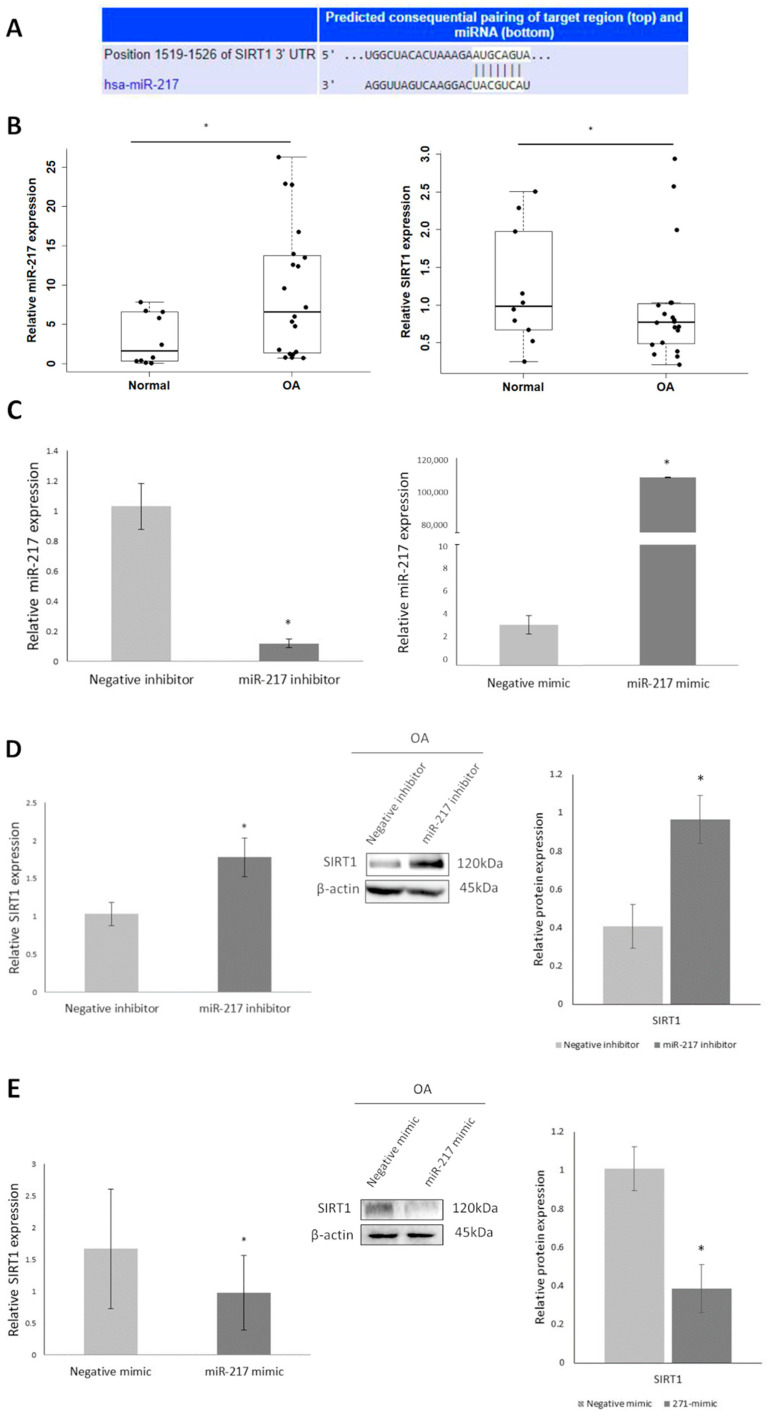
Differential expression of miR-217 affects SIRT1 expression in OA chondrocytes. (**A**) miR-217 seed region targets SIRT1 mRNA with complete complementarity (source; https://www.targetscan.org/vert_80/ accessed on 25 September 2021). (**B**) Differential expression of miR-217-5p and SIRT1 in normal and OA chondrocytes, by qRT-PCR. * *p* < 0.05 (OA vs. normal). (**C**) Differential expression of miR-217-5p in negative inhibitor or miR-217 inhibitor-treated OA chondrocytes, and in negative mimic or miR-217 mimic-treated OA chondrocytes. * *p* < 0.05 (miR-217 inhibitor vs. Negative inhibitor and miR-217 mimic vs. Negative mimic). (**D**) Differential mRNA expression of SIRT1, and representative immunoblot showing differential protein expression of SIRT1 in negative inhibitor or miR-217 inhibitor-treated OA chondrocytes and (**E**) in negative mimic or miR-217 mimic-treated OA chondrocytes. * *p* < 0.05 (miR-217 inhibitor vs. Negative inhibitor and miR-217 mimic vs. Negative mimic).

First, using TargetScan we noticed that the miR-217-5p seed region targets SIRT1 mRNA with complete complementarity (Figure 2A). Given the complete complementarity of miR-217-5p and SIRT1 mRNA target sequence we, then, aimed to address miR-217-5p and SIRT1 expression levels in chondrocytes derived from cartilage of OA patients and healthy donors and found that miR-217-5p was significantly upregulated in OA chondrocytes compared to normal, in contrast to SIRT1 (*p* < 0.05) (Figure 2B). To investigate miR-217-5p/SIRT1 interaction, we treated OA chondrocytes with miR-217 inhibitor and miR-217 mimic and evaluated miR-217-5p and SIRT1 expression levels. MiR-217-5p expression was found significantly downregulated in miR-217 inhibitor-treated OA chondrocytes compared to negative inhibitor-treated cells (*p* < 0.05) and was significantly increased in miR-217 mimic-treated OA chondrocytes compared to negative mimic-treated (*p* < 0.05), confirming the success of transfection (Figure 2C). SIRT1 mRNA and protein expression levels were found significantly upregulated in miR-217 inhibitor-treated OA chondrocytes (*p* < 0.05) (Figure 2D), while a significant decrease was observed in those treated with miR-217 mimic (*p* < 0.05) (Figure 2E), suggesting the impact of miR-217-5p upregulation on SIRT1 inhibition of expression in OA chondrocytes.

### 3.3. MiR-217 Overexpression Affects SIRT1 Deacetylase Activity Promoting the Expression of Inflammatory Markers in OA Chondrocytes

**Figure 3:**
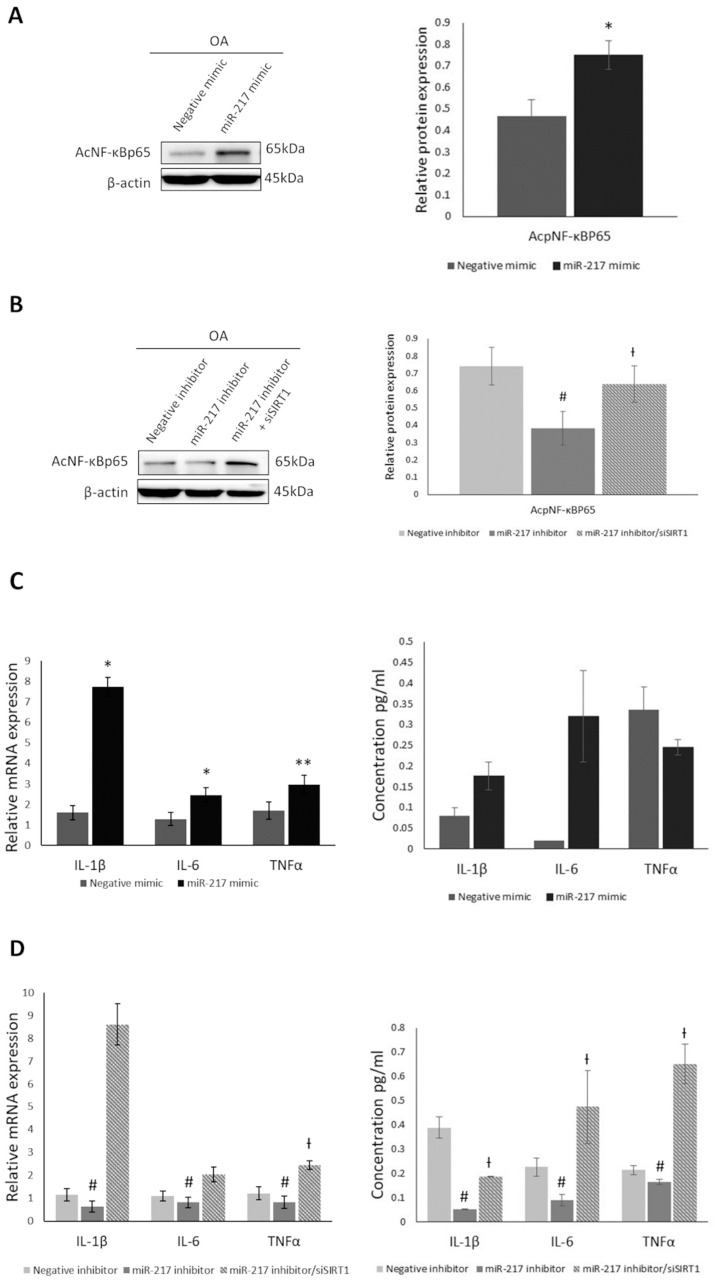
MiR-217 overexpression affects SIRT1 deacetylase activity promoting the expression of inflammatory markers in OA chondrocytes. (**A**) Representative immunoblot presenting differential expression of acetylated NF-Κβp65 in negative mimic or miR-217 mimic-treated OA chondrocytes and (**B**) in negative inhibitor or miR-217 inhibitor ± siSIRT1-treated OA chondrocytes. * *p* < 0.05 (miR-217 mimic vs. Negative mimic), ^#^ *p* < 0.05 (miR-217 inhibitor vs. Negative inhibitor), ^†^ *p* < 0.05 (miR-217 inhibitor vs. miR-217 inhibitor + siSIRT1). (C) Detection of IL-1β, IL-6 and TNFα mRNA expression and secretion levels by qRT-PCR and ELISA, respectively, in negative mimic or miR-217 mimic-treated OA chondrocytes and (D) in negative inhibitor or miR-217 inhibitor ± siSIRT1-treated OA chondrocytes. * *p* < 0.05, ** *p* < 0.01 (miR-217 mimic vs. Negative mimic), ^#^ *p* < 0.05 (miR-217 inhibitor vs. Negative inhibitor), ^†^ *p* < 0.05 (miR-217 inhibitor vs. miR-217 inhibitor + siSIRT1).

To address whether miR-217-mediated impairment of SIRT1 expression affects its deacetylase activity in OA chondrocytes, we initially transfected OA chondrocytes with miR-217 mimic and evaluated SIRT1’s substrate NF-κBp65 [35] acetylation levels. In miR-217 mimic-treated chondrocytes, where SIRT1 expression was found to be significantly decreased, NF-κBp65 acetylation levels showed a significant increase (*p* < 0.05) (Figure 3A). To further investigate the impact of miR-217-mediated SIRT1 downregulation on SIRT1 deacetylation efficiency, we treated OA chondrocytes with miR-217 inhibitor, with or without siRNA against SIRT1 (siSIRT1). siRNA knockdown efficiency of SIRT1 was confirmed by evaluating SIRT1 mRNA and protein expression levels after siSIRT1 (*p* < 0.05) (Supplementary Figure S1). MiR-217 inhibitor-treated cells showed significantly decreased acetylation levels of NF-κBp65 versus the negative inhibitor-treated cells (*p* < 0.05), insinuating a possible association with the restored SIRT1 expression in the miR-217 inhibitor-treated group. In the miR-217 inhibitor/siSIRT1-treated group, where siRNA against SIRT1 counteracted the effect of miR-217 inhibitor treatment on SIRT1 expression, the NF-κBp65 acetylation levels were recovered significantly compared to the miR-217 inhibitor group (*p* < 0.05) (Figure 3B), analogous to miR-217 mimic-treated group results.

Next, to better describe the role of miR-217 mediated SIRT1 suppression on the inflammatory status of OA chondrocytes, we evaluated TNFa, IL-1β and IL-6 mRNA and protein secretion levels, after the aforementioned treatments. As it is known, acetylation of transcription factor NF-κΒ targets these inflammatory markers and induces their expression [35]. The miR-217 mimic-treated group showed a significant increase in TNFα, IL-1β and IL-6 mRNA expression levels versus the negative mimic-treated cells (*p* < 0.05) (Figure 3C). On the contrary, we noticed that in miR-217 inhibitor-treated cells, where NF-κB deacetylation was observed due to SIRT1 expression levels recovery, TNFa, IL-1β and IL-6 mRNA expression levels were significantly reduced (*p* < 0.05) compared to negative inhibitor-treated cells. In the miR-217 inhibitor/siSIRT1-treated group, IL-1β, IL-6 and TNFa mRNA expression levels increased compared to the miR-217 inhibitor group, and significantly for TNFa (*p* < 0.05) (Figure 3D), analogous to miR-217 mimic-treated cells. Regarding TNFa, IL-1β and IL-6 secretion levels, miR-217 mimic-treated group showed an increasing trend for IL-1β and IL-6 compared to negative mimic-treated cells (Figure 3C), while they were significantly reduced (*p* < 0.05) in the miR-217 inhibitor group and recovered in the miR-217 inhibitor/siSIRT1-treated group, in comparison with the miR-217-inhibitor group (*p* < 0.05) (Figure 3D).

All the above suggest that miR-217 overexpression affects SIRT1 deacetylase activity promoting the expression of inflammatory mediators through modulation of NF-KB acetylation levels, which have been characterized as key contributors known to be involved in OA pathogenesis.

### 3.4. MiR-217 Overexpression Affects SIRT1 Deacetylase Activity Enhancing the Expression of Pro-Apoptotic Markers in OA Chondrocytes

**Figure 4:**
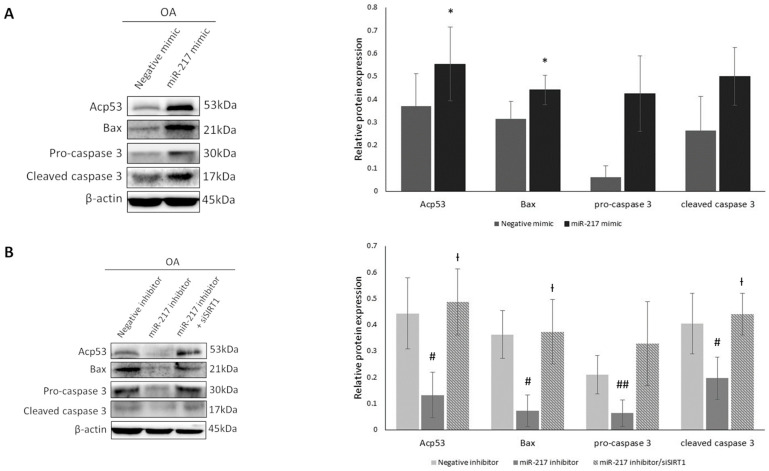
MiR-217 overexpression affects SIRT1 deacetylase activity enhancing the expression of pro-apoptotic markers in OA chondrocytes. (**A**) Representative immunoblot showing differential expression of acetylated p53, Bax, pro-caspase 3 and cleaved caspase 3 in negative mimic or miR-217 mimic-treated OA chondrocytes and (**B**) in negative inhibitor or miR-217 inhibitor ± siSIRT1-treated OA chondrocytes. * *p* < 0.05 (miR-217 mimic vs. Negative mimic), ^#^ *p* < 0.05, ^##^ *p* < 0.01 (miR-217 inhibitor vs. Negative inhibitor), ^†^ *p* < 0.05 (miR-217 inhibitor vs. miR-217 inhibitor + siSIRT1).

To evaluate whether miR-217-mediated inhibition of SIRT1 induces pro-apoptotic genes’ expression, we transfected OA chondrocytes with miR-217 mimic or miR-217 inhibitor ± siSIRT1 and then tested the effects on p53, a robust apoptotic pathway initiator and SIRT1 deacetylation target [36]. For this purpose, we evaluated p53 acetylation levels in miR-217 mimic-treated cells and noticed that they were significantly increased in comparison with negative mimic-treated cells (*p* < 0.05), indicating a possible correlation between miR-217-mediated SIRT1 downregulation and p53 acetylation status (Figure 4A). Conversely, in miR-217 inhibitor-treated cells, p53 acetylation decreased significantly (*p* < 0.05) (Figure 4B). This observation could be related to the observed increase in SIRT1 expression in the miR-217 inhibitor-treated group. In the miR-217 inhibitor/siSIRT1-treated group, p53 acetylation levels were significantly restored compared to the miR-217 inhibitor group (*p* < 0.05) (Figure 4B), confirming the miR-217 mimic-treated group results, supporting, thus, our hypothesis of miR-217 involvement in deacetylase SIRT1′s expression and activity regulation in OA chondrocytes.

In order to further validate the impact of miR-217-mediated SIRT1 suppression on the apoptotic profile of OA chondrocytes, we evaluated protein expression levels of p53 transcription targets and pro-apoptotic markers Bax, pro-caspase 3 and cleaved caspase 3, after performing the above-mentioned treatments. Western blot analysis of the miR-217 mimic group compared to the negative mimic showed an increase, reaching significance only for Bax expression (*p* < 0.05). No significant observations were made regarding the comparison of miR-217 mimic and negative mimic groups for pro-caspase 3 and cleaved caspase 3; however, an increasing trend was observed in both markers’ expression in the miR-217 mimic-treated cells compared to negative mimic-treated OA chondrocytes (Figure 4A). We also found that Bax (*p* < 0.05), pro-caspase 3 (*p* < 0.01) and cleaved caspase 3 (*p* < 0.05) levels were significantly decreased in miR-217 inhibitor-treated OA chondrocytes (Figure 4B). Following subsequent inhibition of SIRT1 expression in the miR-217 inhibitor/siSIRT1-treated group, Bax and cleaved caspase 3 protein expressions were significantly induced compared to the miR-217 inhibitor group (*p* < 0.05). Pro-caspase 3 protein levels were also increased, compared to the miR-217 inhibitor group, but did not reach statistical significance (Figure 4B).

The above results suggest a possible involvement of miR-217-mediated regulation of SIRT1 to the promotion of the above pro-apoptotic markers’ expression, known to induce aberrant apoptotic patterns in OA chondrocytes.

### 3.5. MiR-217 Overexpression Affects the Expression of Matrix Regulating Genes through SIRT1

**Figure 5:**
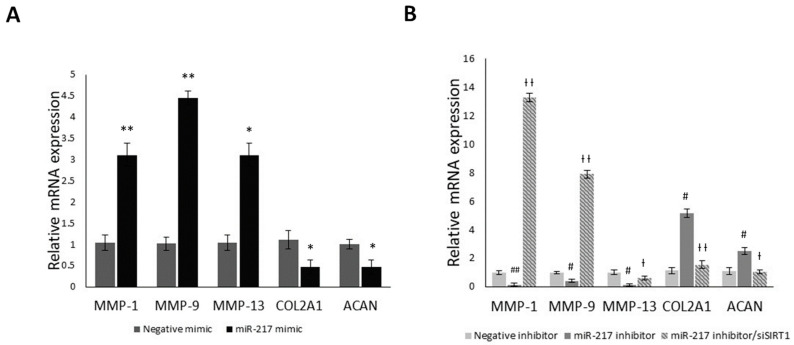
MiR-217 overexpression affects the expression of matrix-regulating genes through SIRT1. (**A**) Differential mRNA expression levels of MMP-1, MMP-9, MMP-13, COL2A1 and ACAN in negative mimic or miR-217 mimic-treated and (**B**) in negative inhibitor or miR-217 inhibitor ± siSIRT1-treated OA chondrocytes, by qRT-PCR. * *p* < 0.05, ** *p* < 0.01 (miR-217 mimic vs. Negative mimic), ^#^ *p* < 0.05, ^##^ *p* < 0.01 (miR-217 inhibitor vs. Negative inhibitor), ^†^ *p* < 0.05, ^††^ *p* < 0.01 (miR-217 inhibitor vs. miR-217 inhibitor + siSIRT1).

It has been previously shown that SIRT1 regulates various ECM components such as COL2A1 and ACAN [37], as well as cartilage matrix degrading/catabolic genes such as MMP-1, MMP-9 and MMP-13 [35,38]. In that regard, we aimed to evaluate their mRNA expression levels in miR-217 mimic or miR-217 inhibitor ± siSIRT1 treated OA chondrocytes (Figure 5). A significant increase in all three MMPs was observed (*p* < 0.05) in the miR-217 mimic group, where SIRT1 expression was decreased, while COL2A1 and ACAN mRNA expression levels were significantly downregulated, compared to negative mimic-treated OA chondrocytes (*p* < 0.05) (Figure 5A). However, in miR-217 inhibitor-treated OA chondrocytes, where increased SIRT1 expression was observed, MMP-1, MMP-9 and MMP-13 expression levels were significantly reduced compared to the negative inhibitor-treated group (*p* < 0.05) (Figure 5B). This effect was reversed following siSIRT1 transfection, with significant restoration of all three catabolic markers’ mRNA expression levels (*p* < 0.05). On the contrary, COL2A1 and ACAN mRNA expression levels were significantly recovered in accordance with the observed SIRT1 upregulation, while in miR-217 inhibitor/siSIRT1-treated OA chondrocytes, their expression levels dropped significantly (*p* < 0.05) (Figure 5B).

The presented results insinuate that miR-217-5p could, possibly, have an indirect impact on the expression of cartilage ECM regulating genes through targeting SIRT1, which expands beyond its pro-inflammatory and pro-apoptotic effects in OA chondrocytes.

## 4. Discussion

MiR-217 has been identified as a crucial regulator of cellular senescence [16,17] and exhibits a key role in stress-induced inflammation and apoptosis-related pathways in age-related diseases [22]. Thus, we aimed at elucidating miR-217 impact on deacetylase’s SIRT1 expression and activity levels in OA chondrocytes.

At first, using three (3) public databases, TargetScan, miRDB and DIANA, we extracted miR-217 predicted target genes. Next, GO and KEGG pathway analyses showed that miR-217 targets were mainly involved in cell senescence and longevity pathways, regulation of gene expression, protein/histone deacetylation, regulation of programmed cell death and oxidative stress-induced apoptotic pathways. Sorting out only the experimentally validated miR-217 mRNA targets, we used the Open Targets platform to address the ones involved in OA. *SIRT1* was selected as the most robust and validated miR-217 target gene involved in OA pathology. SIRT1 has been previously characterized as a central regulator of aging in pathological manifestations associated with age [31,32], while it has also been found to exhibit significantly impaired expression in OA tissues, which weakens its chondroprotective effect on OA chondrocytes [33,34,37,38,39,40]. In that regard, we initially hypothesized that miR-217 might be involved in SIRT1 expression regulation in OA chondrocytes.

To test this hypothesis, we proceeded by evaluating miR-217 expression in normal and OA chondrocytes and found that miR-217 was significantly upregulated in OA chondrocytes compared to normal. Having found SIRT1 mRNA complete complementarity with miR-217, we implied miR-217 negative correlation with SIRT1 low expression in OA chondrocytes. MiR-217 expression in human OA and normal chondrocytes has been previously reported in a recent study by Liu et al. [41], who showed that miR-217 expression is reduced in OA cartilage compared to normal. However, the chondrocytes used in the Liu et al. study were in passage 3, while in our study we used passage 0–1 chondrocytes. As it is known that chondrocyte passaging can cause chondrocytes’ dedifferentiation and loss of phenotype [42], the increased passages may be a possible reason for the contradicting results between Liu et al. [41] and the present study, regarding miR-217 expression in OA vs. normal chondrocytes.

To elucidate the interaction of miR-217 and SIRT1, we treated OA chondrocytes with miR-217 inhibitor or miR-217 mimic and evaluated miR-217 and SIRT1 expression. We observed that miR-217 inhibition significantly restored SIRT1 mRNA and protein expression levels. On the contrary, miR-217 mimic treatment resulted in significant reversion in SIRT1 expression, in line with our initial hypothesis.

Chondrocytes exhibiting reduced SIRT1 levels have been found to present a potent inflammatory phenotype accompanied by increased catabolic activity, due to the pro-inflammatory factors TNFa and IL-1β contributing, thus, to articular cartilage degradation as they result in aberrant expression patterns of cartilage ECM degrading enzymes, such as MMP-1, MMP-13, MMP-9 and ADAMTS-5 [35,39,43,44], as well as in deficiency of matrix components like type II collagen and aggrecan [37]. These cytokines show high levels of expression in almost all joint tissues (cartilage, synovial membrane and subchondral bone) in OA patients, while they can act individually and/or simultaneously with other mediators of inflammatory response to initiate and enhance the sequence of inflammatory events [45]. NF-κB activation by inflammatory cytokines not only induces catabolic gene transcription but also stimulates inflammatory mediators such as TNFa, IL-1β and IL-6 through a positive feedback loop [45,46,47]. NF-κΒ is a SIRT1 deacetylation target, specifically in Lys310 of RelA/p65 subunit, alleviating inflammatory manifestations in OA cartilage [35,48,49,50]. Concurrently, one of SIRT1’s main targets is transcription factor p53, involved in apoptosis, cell cycle arrest and aging. SIRT1 deacetylates p53 at its C-terminal lysine residue K382, inhibiting its transcriptional activity, and contributing to the inhibition of cellular aging [36]. Due to its deacetylase enzymatic capacity, SIRT1 has a well-characterized anti-apoptotic role in chondrocytes. Inhibition of SIRT1 has been associated with increased levels of pro-apoptotic Bax and concomitant reduction in anti-apoptotic Bcl-2 molecules that favor chondrocyte survival [49]. SIRT1 contribution to chondrocyte apoptosis appears to be mediated by the activation of caspases 3 (effector caspase) and 9 (initiator caspase) acting mainly through the endogenous pathway where mitochondria play a key role [50].

Based on the above, we aimed to investigate whether miR-217-mediated SIRT1 downregulation also affects SIRT1 deacetylase anti-inflammatory and anti-apoptotic activity in OA chondrocytes. We observed that upon SIRT1 downregulation in miR-217 mimic-treated OA chondrocytes, NF-κΒp65 exhibited increased acetylation, as opposed to miR-217 inhibitor treatment which subsequently led to decreased NF-κBp65 acetylation levels, due to SIRT1 increase. In addition, SIRT1 downregulation in miR-217 mimic-treated OA chondrocytes enhanced NF-κΒ’s transcriptional activity on specific downstream target genes with a key role in inflammation, as declared by the induced levels of TNFa, IL-1β and IL-6. Furthermore, inhibition of miR-217 affected the apoptotic/survival profile of OA chondrocytes, mainly via SIRT1-mediated p53 deacetylation resulting in decreased expression of the pro-apoptotic proteins Bax and cleaved caspase 3. Finally, miR-217 upregulation resulted in increased MMP-1, MMP-9 and MMP-13 expression, while it also affected the expression of COL2A1 and ACAN, two key structural components of cartilage ECM [37,51]. Our novel reported findings show that miR-217 overexpression in OA chondrocytes contributes to SIRT1 downregulation, impairing its chondroprotective effects against catabolic, inflammatory and pro-apoptotic responses that lead to cartilage degeneration.

Although miR-217/SIRT1 interaction has not been previously examined in OA chondrocytes, it has been reported in the literature regarding some prevalent age-related pathological/morbid conditions such as CVD, hypertension, atherosclerosis, diabetes, neurodegenerative diseases, arthritis and cancer [27]. For example, miR-217 has been found to be the highest induced miRNA in endothelial cell senescence and dysfunction in CVD, where overexpression of miR-217 induced vascular endothelial cell senescence by targeting the SIRT1/p53 signaling pathway in human umbilical vein endothelial cells (HUVECs) [16]. MiR-217 has also been associated with macrophage apoptosis in endothelium-specific knock-in mouse models for atherosclerosis, where it was reported to downregulate SIRT1 in vivo [17].

The findings of this study suggest that miR-217 overexpression results in SIRT1 downregulation and evokes catabolic, inflammatory and apoptotic manifestations in OA chondrocytes. As miR-217 has been shown to sponge different circRNAs, such as circ-VANGL1 and promote osteoporosis development by downregulating RUNX2 expression, or circRNA_100367 [52,53] it would be interesting that future studies explore circRNAs/miR-217 interactions and SIRT1 regulation in OA chondrocytes. In the meantime, as miRNA-mediated regulation of gene expression has become an emerging field, especially in anti-cancer research, anti-miR compounds and specific miRNA inhibitors could be revealed as a new potential class of drugs. In that regard, future relative in vitro/in vivo studies using selective miR-217 inhibitors might demonstrate miR-217 inhibition targeting SIRT1 as a potential therapeutic protocol against OA progression.

## 5. Conclusions

Our study provides novel evidence implying that miR-217-mediated SIRT1 downregulation in OA chondrocytes results in increased NF-κB and p53 acetylation, proposing that modulation of miR-217-5p expression could affect the expression of pro-inflammatory, pro-catabolic and pro-apoptotic responses in OA chondrocytes. Our results emphasize the importance of miRNA regulation for the potential of designing new therapeutic strategies against OA.

## Figures and Tables

**Table 1:** (**a**) Stem-loop primer sequences and (**b**) primer sequences for qRT-PCR.

(**a**) Stem-loop primer sequences		
U6 stem-loop	5′-CACGGAAGCCCTCACACCGTGTCGTTC-3′	
miR-217-5p stem-loop	5′-GTCGTATCCAGTGCAGGGTCCGAGGTATTCGCACTGGATACGACTCCAAT-3′	
(**b**) qRT-PCR primer sequences		
**Gene Name**	**Forward Primer (5′** **-** **3′)**	**Reverse Primer (5′** **-** **3′)**
U6	GCTTCGGCAGCACATATACTAAAAT	CTCACACCGTGTCGTTCCA
miR-217-5p	CGCTCTACTGCATCAGGAACTGA	GTGCAGGGTCCGAGGT
GAPDH	GAGTCAACGGATTTGGTCGT	GACAAGCTTCCCGTTCTCAG
SIRT1	CTGCCTGGATCCCCTTAGTT	TATAATCAGGGGCCTGTTGC
IL-1β	ATGGACAAGCTGAGGAAGATG	CCTCGTTATCCCATGTGTCG
IL-6	CAACCTGAACCTTCCAAAGATG	ACCTCAAACTCCAAAAGACCAG
TNFα	CCTGAAAACAACCCTCAGA	AAGAGGCTGAGGAACAAGCA
MMP-1	ACGTTCCCAAAATCCTGTCC	GGGTAGAAGGGATTTGTGCG
MMP-9	TTGACAGCGACAAGAAGTGG	GCCATTCACGTCGTCCTTAT
MMP-13	TGGCATTGCTGACATCATGA	GCCAGAGGGCCCATCAA
COL2A1	ATGACAATCTGGCTCCCAACACTGC	GACCGGCCCTATGTCCACACCGAAT
ACAN	TGAGGAGGGCTGGAACAAGTACC	GGAGGTGGTAATTGCAGGGAACA

## Data Availability

The data presented in this study are contained within the article and/or are available in Supplementary Materials.

## Supplementary Materials

The following supporting information can be downloaded at: https://www.mdpi.com/article/10.3390/genes14122155/s1, Figure S1: siRNA against SIRT1 knockdown efficiency; Table S1: GO biological process and KEGG pathways enrichment analyses full outputs.
