# Supplementary material for: MiR-217 Regulates SIRT1 Expression and Promotes Inflammatory and Apoptotic Responses in Osteoarthritis
Source: Genes (Basel). 2023 Nov 29;14(12):2155. doi: 10.3390/genes14122155 (PMC10742866; doi:10.3390/genes14122155)
Supplement: Supplementary file 1 [file genes-14-02155-s001.zip › Supplementary Figure S1.pdf]

A

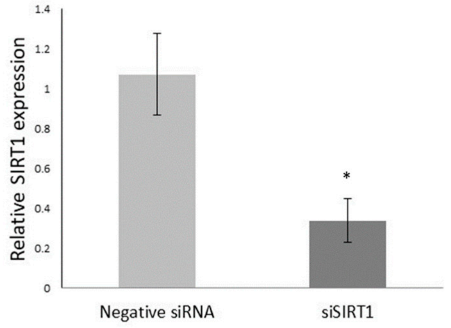

B

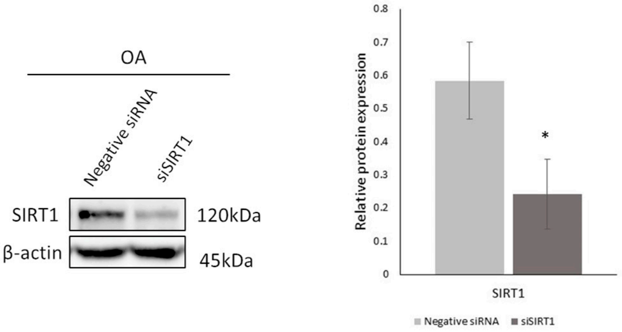

Supplementary Figure S1: siRNA against SIRT1 knockdown efficiency. (A) Differential mRNA expression of SIRT1 in siRNA Negative Control-treated and siSIRT1-treated OA chondrocytes analyzed by qRT-PCR and normalized against GAPDH expression. *$p<0.05$ (siSIRT1 vs siRNA Negative Control). (B) Representative immunoblot showing differential protein expression of SIRT1 in siRNA Negative Control-treated and siSIRT1-treated OA chondrocytes. Antibody against β-actin was used as loading control. *$p<0.05$ (siSIRT1 vs siRNA Negative Control).
